# Translation of a community palliative care intervention: Experience from West Bengal, India

**DOI:** 10.12688/wellcomeopenres.14599.1

**Published:** 2018-05-31

**Authors:** Devi Vijay, Shahaduz Zaman, David Clark

**Affiliations:** 1 Organizational Behavior Group, Indian Institute of Management Calcutta , Kolkata, West Bengal, 700104, India; 2 Global Health and Infection Department, Brighton and Sussex Medical School, University of Sussex, Brighton, BN1 9PX, UK; 3 School of Interdisciplinary Studies, University of Glasgow, Dumfries, DG1 4ZL, UK

**Keywords:** Community-based organizations, palliative care, translation, India

## Abstract

**Background**: The community form of palliative care first constructed in Kerala, India has gained recognition worldwide. Although it is the subject of important claims about its replicability elsewhere, little effort has gone into studying how this might occur. Drawing on translation studies, we attend to under-examined aspects of the transfer of a community palliative care intervention into a new geographic and institutional context.

**Methods**: Over a period of 29 months, we conducted an in-depth case study of Sanjeevani, a community-based palliative care organization in Nadia district, West Bengal (India), that is modelled on the Kerala approach. We draw upon primary (semi-structured interviews and field notes) and secondary data sources.

**Results: **We identify the translator’s symbolic power and how it counteracts the organizational challenges relating to socio-economic conditions and weak histories of civil society organizing. We find that unlike the Kerala form, which is typified by horizontal linkages and consensus-oriented decision-making, the translated organizational form in Nadia is a hybrid of horizontal and vertical solidarities. We show how translation is an ongoing, dynamic process, where community participation is infused with values of occupational prestige and camaraderie and shaped by emergent vertical solidarities among members.

**Conclusions**: Our findings have implications for how we understand the relationship between locations, institutional histories, and healthcare interventions. We contribute to translation studies in healthcare, and particularly to conversations about the transfer or ‘roll out’ of palliative care interventions from one geography to another.

## Introduction

This study examines the translation of a community-based palliative care intervention into a new institutional context. Community-based healthcare approaches have been widely recognized since the Alma Alta Declaration in 1978, which emphasized the bottom-up role of communities in improving their health status and reaching a state of physical, mental, and social well-being ( Abel *et al.*, 2013; Bardach *et al.*, 2017; Draper *et al.*, 2010; Rifkin, 1986; World Health Organization, 1978). Yet, even when effective ideas are identified, they seldom travel between contexts, and have variable and often disappointing results when replicated ( Lawrence, 2017). Indeed, public health researchers increasingly highlight the problems of uncritical policy and practice transfer, emphasizing instead the *translation* of interventions (e.g. Kothari & Armstrong, 2011; Margaret Dolcini *et al.*, 2010; Mannell, 2014; Montesanti *et al.*, 2015). Unlike studies of replication, diffusion, or transfer that denote actors as passive recipients of ideas, translation approaches emphasize context and actors’ reflexivity ( Czarniawska & Joerges, 1996; Sahlin & Wedlin, 2008).

Organizational forms have a direct bearing on an intervention’s adoption and effectiveness in the target setting ( Tracey *et al.*, 2018). We define organizational forms as “archetypal configurations of structures and practices that are given coherence by underlying values, and that are regarded as appropriate within an institutional context” ( Greenwood & Suddaby, 2006, p.30). Accordingly, a community form is a distinct organizational configuration typified by consensus-building practices, and non-hierarchical member relations and values ( Kothari & Armstrong, 2011; Marquis *et al.*, 2011). Research informs us of the strategic challenges in translating community forms, such as establishing community partnerships, gathering local information, and transforming frameworks for local adoption (e.g. Draper *et al.*, 2010; Kothari & Armstrong, 2011; Montesanti *et al.*, 2015). However, we rarely attend to the construction of the new organizational form during translation, and the social, political and historical conditions that shape this translation process.

We examine the translation of a community-based palliative care intervention into Nadia district, West Bengal, India. Palliative care is an approach to improving the quality of life of patients and families with life-limiting conditions ( World Health Organization, 2017). Community-based care which emerged in Kerala, India has received widespread attention as a contextually appropriate, resource-effective alternative to hospital and hospice-based approaches to palliative care ( Kumar, 2013; Quality of Death Report, 2010; Quality of Death Report, 2015). Three key practices characterize the community approach. First, community members – such as farmers, bus drivers, teachers - are the locus of care-giving, decision-making and fund-raising, with medical professionals providing need-based support. Second, palliative care is conceptualized as ‘total care’, including medical, social, and financial support, rehabilitation and bereavement services for patients and families. In this manner, over two decades, Kerala has expanded palliative services to a wide-range of diagnostic groups including cancer, chronic cardiovascular and renal conditions, mental health issues, geriatric needs, and paraplegia. Third, this model involves integrating community organizations into public health systems wherein they collaborate with local self-governing institutions for care delivery. A measure of the success of the model was seen in 2008, when in response to bottom-up advocacy by community organizations, the Kerala state government passed a landmark policy mandating that state-funded primary health centres be equipped with professionals trained in palliative care and resourced with locally required opioids ( Kumar, 2013; Vijay & Kulkarni, 2012).

In these ways, the Kerala palliative care movement has mobilized over 15000 volunteers, 65 physicians, and 180 nurses in over 200 community organizations, to cover more than 60% of the patient population across all 14 districts of Kerala, making it the only Indian state to have palliative care in every district ( Quality of Death Report, 2015). In sharp contrast, coverage in the rest of India (where palliative care is predominantly provided through hospices or hospitals) is less than 2–3%, with most states having no known palliative care provision ( McDermott *et al.*, 2008; Rajagopal, 2015). The large scale and scope of Kerala’s palliative care model has strengthened global discourse on community-based palliative care interventions as a public health strategy (e.g. Abel *et al.*, 2013; Sallnow *et al.*, 2012), with attempts to replicate it not only in India, but also in Bangladesh, Switzerland, Sri Lanka, and Thailand. Simultaneously, questions have been raised about the transferability of the Kerala model to other institutional contexts ( Sallnow *et al.*, 2010).

Three key findings emerge from our study. First, our institutional analysis delineates the organizational challenges posed by socio-economic conditions and weak institutional histories of civil society organizing. The translator’s employment of symbolic power legitimates the nascent community form in this institutional context. Second, unlike the community form in Kerala typified by horizontal linkages, the community form in Nadia emerges as a *hybrid* of vertical and horizontal solidarities. Third, translation here is as an ongoing, dynamic process, infused with values of occupational prestige and camaraderie and shaped by emergent vertical solidarities. Such an understanding of translation processes adds texture to discussions on the transfer and ‘roll out’ of best practices and frameworks beyond the institutional contexts in which they originate ( Abel *et al.*, 2013; Montesanti *et al.*, 2015).

## Theoretical framework

We define translation as the selective interpretations of new ideas, practices, or forms that are contextually appropriate or may involve “local adaptations and transformations” ( Zilber, 2006, p.300). Accordingly, ideas do not diffuse in a vacuum, and actors are not simply passive recipients of new practices. Rather, translation studies attend to how ideas and practices travel and are transformed from one institutional context to another through actors’ interactive, negotiated processes ( Czarniawska & Joerges, 1996; Hwang & Suarez, 2005). Translation is therefore a doubly contextualized process, reflecting both the historical and cultural conditions within which an idea emerges, and the conditions to which it is transposed ( Wolf & Fukari, 2007). To translate, then, is to construct anew, as variations take on different forms and meanings in different institutional contexts ( Czarniawska & Joerges, 1996; Sahlin & Wedlin, 2008).

Power and domination underpin the act of translation, as the translator’s social position and prestige influence her visibility (or invisibility) and acceptability ( Wolf & Fukari, 2007). Bourdieu develops the theory of domination by distinguishing between different species of capital. Symbolic capital is a specific form of capital commonly designated as prestige and authority. Symbolic power then arises out of symbolic capital as the belief “by virtue of which persons wielding authority are endowed with prestige” ( Bourdieu & Wacquant, 2013, p. 299).

The translation of a new form into a distinct institutional context presents unique challenges compared to introducing it to a similar institutional context, or translating subsidiaries, chapters, or franchises of an existing organization ( Tracey *et al.*, 2018). Additionally, translation into a new institutional context presents specific challenges in the case of community organizations. First, it is problematic to assume the *a priori* presence in the target field setting of a cohesive community with shared purpose. While actors may recognize mutual interests and a potential for shared organization, this does not necessarily translate into coordinated and effective action ( Hardy, 1994). Thus, not only must an idea travel across boundaries, but transferring a community form also involves multiple actors coming together to co-ordinate and engage in productive work. Second, community forms are marked by specific organizing processes that are shaped by geographies and the histories of organizing ( Greve & Rao, 2014; Marquis *et al.*, 2011). Accordingly, communities vary in their institutional infrastructure – such as legal infrastructure, presence of other voluntary organizations, and intra-community relations – for collective action ( Greve & Rao, 2012; Greve & Rao, 2014). Finally, even after a community form is seeded, organizing may be reduced to a few individuals’ volunteerism, and collective momentum may not be maintained ( Aveling *et al.*, 2012). How then are community-based healthcare organizations translated across distinct institutional contexts?

## Empirical context

Our study is set in West Bengal, a state in eastern India. Nadia, the focus geography of our study, has a population of 5,167,600 (~5.66% of West Bengal’s population) and is one of 21 administrative districts of West Bengal. As with the rest of India, healthcare in West Bengal is largely privatized and accessed through out-of-pocket expenses ( Garg & Karan, 2008). The Sanjeevani Palliative Care Society (henceforth, Sanjeevani), inaugurated on 22 ^nd^ September 2014, was the first experiment in community mobilization for palliative care in West Bengal, and among the few nascent experiments outside Kerala. Sanjeevani (meaning “life-giving”) was spearheaded by the District Magistrate of Nadia, in collaboration with physicians from the local chapter of the Indian Medical Association, and the Institute of Palliative Medicine (IPM), Kerala. Sanjeevani was registered as a non-government organization. Three patient categories in Nadia were identified for inclusion, those with: advanced cancer, chronic renal conditions, and the chronically bed-ridden. Non-communicable diseases account for more than 52% of illnesses within Nadia ( Pratichi Report, 2012). Patients presented at advanced stages of chronic and life-threatening conditions, rendering the need for palliative care services more immediate and essential ( McDermott *et al.*, 2008).

## Research methods

We combine primary and secondary analysis in this study. The research team has been involved in different ways in studying Kerala’s community form emergence and its translation to Nadia. We thus used our previous research findings and publications, other published sources collected though ongoing monitoring of the specialist literature, and reflections from our engagement in Kerala and Nadia - to inform our analysis ( Aveling *et al.*, 2017; Campbell & Cornish, 2012). Specifically, we drew on our past research on the Kerala model (see Graham & Clark, 2005; Vijay, 2012; Vijay & Kulkarni, 2012; Zaman *et al.,* 2017). We also searched the specialist literature for articles that specifically referenced palliative care in Kerala. This yielded 116 articles that comprised our dataset ( Supplementary File 1). This secondary data informed our understanding of contextual differences, and trajectories of evolution of palliative care in Kerala.

We drew upon primary data from field observations, interviews, and personal communications between various actors in Nadia. Our study spanned 29 months (June 2014-November 2016), commencing with participation in volunteer trainings prior to the official founding of Sanjeevani, and continued until we reached empirical and theoretical saturation ( Erlandson *et al.*, 1993). Ethics approval was obtained from the University of Glasgow, College of Social Sciences, with which two authors are affiliated (approval number, 400150044). Verbal informed consent was obtained from participants since written consent is difficult to obtain in subaltern settings where participants have varying literacy levels. In previous research, we have seen that signing any document is treated with suspicion in subaltern contexts, especially since we were introduced to the setting through a government official. Twenty two participants were sampled purposively for semi-structured interviews to understand different stakeholders’ perspectives. Accordingly, interviews were conducted with the District Magistrate, physicians of the local Indian Medical Association, volunteers and nurses, and visiting members of IPM. Interviews (lasting 20–90 minutes) were in English (4) and Bengali (18) and were audio-recorded. We conducted eight field visits, involving attendance at awareness workshops, training sessions, review meetings, and Sanjeevani’s foundation day. In total, Krishnagar municipality and six of 30 panchayats (panchayat is a village council with elected representatives) were visited. Home care teams were accompanied during 40 patients’ home visits. These field visits enabled observation of participants’ interactions, collective dynamics, and the emergence of tensions, at various stages. Multiple interactions with patients and families occurred during home care visits.

Analysis proceeded through various stages alongside data collection. First, analysing institutional legacies that shape communities ( Greve & Rao, 2014), we distinguished between the institutional contexts in Kerala and West Bengal. Second, developing a thick description of the Sanjeevani case, we identified aspects of the community form (in terms of practices and relational structures) that materialized in Nadia. Finally, we used constant comparative analysis to identify major themes ( Strauss & Corbin, 1990). At this stage, we identified resource mobilization, low-levels of legitimacy, top-down organizing, teams and team leaders, occupational prestige and camaraderie, as salient themes. Iterating between theory and data, we clustered these themes into interpretive categories relevant to the translation process. As per Erlandson *et al*. (1993), we established trustworthiness of our findings through the triangulation of data sources (e.g. diverse stakeholders), multiple methods (interviews across different time periods, observations), and researcher triangulation. By written agreement, we use participants’ real names in cases where their identities are known in the public domain (e.g. District Magistrate of Nadia or Director of the Institute of Palliative Medicine), or where participants’ views had been reported in media sources. We used pseudonyms (indicated with an asterisk) for all other participants to maintain anonymity and confidentiality.

## Institutional context for community-based organizing in Kerala and West Bengal

We focused our analysis of the institutional contexts of Kerala and West Bengal by examining the institutional legacies of voluntary organizations, intra-community relations, and legal structures pertaining to healthcare ( Greve & Rao, 2014). We also compared health infrastructure in both contexts. Table 1 provides a summary.

**Table 1.** Differences in institutional context.

	Kerala	West Bengal
1. Demographic indicators		
a. Literacy *	93.91%	76.26% - In Nadia - 74.97%
b. Life expectancy *	73.2% Male 77.6% Female	69.2% Male 72.1% Female
c. Infant mortality rate *	12	32
d. Maternal mortality ratio *	81	145
*e. State Government’s per capita* health expenditure ** (in Rupees, *2009–2010)*	499	330
*f. % Population below poverty line* (2012) ***	8%	20%
2. Legal infrastructure for palliative care		
*a. Narcotics and Psychotropic* *Substances Act*	Amended in 1999 to ease opioid access for pain relief	Amended in 2014 to ease opioid access for pain relief - Access on the ground remains limited. Sanjeevani had no access to morphine.
*b. State Government palliative care* *policy*	In 2008, Kerala state mandated provision of palliative care at primary health center level	No equivalent policy
3. Voluntary organizations	High density of civil society organizations, including community organizations, co- operatives	Patron-client relations with political parties; political mobilization of communities

* Census, 2011 ** Choudhury & Nath, 2012 *** World Bank Group, 2012

Kerala presents a unique socio-political and historical context for a community form. The state is characterized by high levels of literacy and health indicators (see Table 1) comparable with many developed countries ( India Human Development Report, 2011). Moreover, Kerala has the highest per capita expenditure on health care in India ( Jeffrey, 2011). These social indicators have been attributed to a vibrant civil society (characterised by a high density of co-operatives and community organizations) and a history of mobilizing for rights, especially in healthcare ( Santhosh, 2016; Vijay, 2012). Such social structures decrease the costs of collective action and enhance shared logics of action and opportunities for resource mobilization ( Greve & Rao, 2014). Further, for decades, the Kerala government has invested in health infrastructure with robust, functioning public health facilities and there have been numerous experiments with community organizations in healthcare ( Jeffrey, 2011). Thus, a community approach, albeit novel to the palliative care field, was a legitimate organizational template in Kerala.

In contrast, West Bengal has weaker histories of community-based organizing and institutional infrastructure for healthcare. Studies point to Bengal’s ‘party-society’ logic, characterized by patron-client relationships between the Bengali population (especially in rural areas) and the two regionally dominant political parties ( Bhattacharya, 2009; Roy, 2012). An outcome of this clientalism is that political parties tend to displace other competing channels of public transactions such as civil society organizations ( Bhattacharya, 2009).

Nadia district, where the Sanjeevani experiment unfolds, is predominantly a rural region. Patients we tracked in Nadia district typically travelled to Kolkata (the capital city of West Bengal, approximately 150 kilometers from Krishnagar - the Nadia district headquarters) for treatment. Having done field work in different parts of Kerala and in Nadia, we observed the stark destitution and lack of access to basic healthcare facilities at Nadia. Patients’ families were typically engaged in agriculture, masonry, and similar forms of manual wage labour. Several availed of the government funded Mahatma Gandhi National Rural Employment Guarantee Act Scheme that guarantees 100 days of work per year. Patients with curable conditions were deemed incurable by family members, due to poor health literacy, misinformation from middle-men, and economic difficulties of accessing robust healthcare. Thus, we see Sanjeevani germinate in a vastly different socio-political context from the source context in Kerala.

## Key moments of translation

We identified three moments that influenced the translation of community-based palliative care practices to Nadia: the translator’s enactment of symbolic power, translating member relations, and translating social participation.

### Translator’s enactment of symbolic power

***Enabling resource mobilization.*** The District Magistrate (DM), P.B. Salim played a pivotal role in crafting Sanjeevani as a community form in Nadia. Upon taking office at Nadia in March 2014, Salim invited Suresh Kumar, a pioneering palliative care physician and the founding Director of the Institute of Palliative Medicine, Kerala, to hold an initial core meeting with local stakeholders (including physicians, activists and volunteer social workers) Salim had identified. With his knowledge of over 25 years of community-based organizing, Suresh observed:

This involvement [of the DM] resulted in a faster mobilization of resources, as compared to what CBOs [community-based organizations] grapple with in Kerala, as community volunteers spend a considerable amount of time in the first year seeking funding and core volunteers, besides seeking legitimacy for their efforts

A DM’s position is that of a “mini-monarch” within the Indian Administrative Services hierarchy, with jurisdiction over almost 50 departments including agriculture, law and order, revenue, welfare and development ( Bandyopadhyay, 2006, p. 4851). Since DMs typically spend a considerable amount of time dealing with immediate, every-day issues of law and order and public grievances, they are less likely to be involved in long-term development and welfare projects. However, Salim’s welfare projects for marginalized populations during previous official deputations had earned him the epithet of “People’s Collector” (Collector is another title for District Magistrate). At Nadia, prior to the launch of Sanjeevani, he initiated the Sabar Shouchagar Project (‘Toilet for Everyone’), transforming Nadia into India’s first open defecation-free district ( Vijay & Ghosh, 2018) and subsequently winning the United Nations Public Services Award 2016.

Thus, Salim’s involvement with Sanjeevani garnered considerable attention, in addition to media coverage. The initial awareness program saw the enrollment of over 1000 participants from diverse backgrounds, such as students, home-makers, teachers, and local social workers, who attended the kick-off meeting in May 2014. Thereafter awareness camps and training programmes were held in public auditoriums, such as the Zilla Parishad Bhavan. Sanjeevani’s launch in September 2014 was a grand function at the district auditorium, attended by more than a dozen elected political representatives. At the time of inauguration, Sanjeevani had the initial support of 14 physicians and two nurses (professional resources that are acutely short in India), along with over 150 master trainers and 1000 trained community volunteers. Master trainers trained other interested volunteers in their own neighborhoods. Two ambulances were donated by local elected political representatives.

***Overcoming low legitimacy.*** Most of our participants had not heard of the Kerala model prior to its introduction in Nadia. Additionally, during training programs and awareness workshops, participants raised doubts about whether such an initiative would materialize in West Bengal. For instance, at a training, a participant openly stated, *“in Bengal we have a political society, not a civil society. I am not sure this will work”*. Such articulations were tethered to views that the institutional context was not conducive to a civil society organization without any political party affiliation.

During pilot home visits to survey potential patients’ needs, volunteers were interrogated by family members on which political party they represented. In the absence of a political affiliation, families expressed skepticism about the volunteers’ intentions. A physician shared concerns about this lack of legitimacy for non-governmental organizations (NGOs):

A lot of NGOs have laundered money with malpractices. People do not hold high regard for NGOs, because, in general, they are inclined towards corruption and malpractices. We wanted to steer clear of an NGO name, but then for registration we need that.

In one case, a patient’s family expected economic support and did not comprehend the need for home-based palliative care, insisting that Sanjeevani provide financial aid for the patient’s treatment. When no financial aid was in sight after a few visits, the family warned the team that *"don't ever come back to our house"* ( *Tomra ar ashbe na amader barite*). Such harsh encounters in the early stages created anxieties among volunteers regarding their work and Sanjeevani’s viability.

Under such circumstances, the DM’s affiliation played a vital role in signaling and legitimating Sanjeevani’s efforts. Sanghamitra was a palliative care physician working independently in Kolkata. She had experienced first-hand the difficulties of finding volunteer and nursing support. Sanghamitra provided initial support for one year to Sanjeevani and recognized the symbolic power of the DM’s involvement in attracting volunteers and patients. She observed: “ *The District Magistrate was involved. People thought it has to be big, and it has to be something to do with good work*”.

Thus, Salim’s symbolic capital was manifest as Sanjeevani took root. Towards the end of Salim’s tenure in Nadia and impending relocation to another district, our participants were anxious about Sanjeevani’s sustainability in Salim’s absence and shared anxieties over his successor. After his tenure, Salim continued to visit Sanjeevani on occasion, during which he helped team members address pressing challenges, such as finances. Sanjeevani members explicitly articulated that Salim’s presence and periodic visits were pivotal to their motivation and the initiative’s sustainability. We thus contend that in this distinct institutional context, the translator’s symbolic power was significant in mobilizing the necessary conditions of social interaction to realize the intervention ( Wolf & Fukari, 2007).

### Hybridizing member relations: Emergent vertical and horizontal solidarities

***Top-down organizing.*** At Nadia, the DM mobilized a coalition comprising local social activists and physicians from the Indian Medical Association’s local chapter. This coalition became Sanjeevani’s core committee, driving further activities. IPM, a World Health Organization Collaborating Center, provided specific inputs on the technical and organizational aspects from the earliest training phases at Nadia. After the official launch, IPM members visited Sanjeevani bi-monthly for the first six months, and then quarterly, accompanying home-care teams, and participating in core committee meetings, trainings, and awareness programs.

Revealing their social distance, community volunteers referred to the DM and physicians with honorifics such as “Honourable”, “Sir”, with one volunteer sharing that, “ *It feels as though they [physicians] are helping us like Gods would. We have their blessings. They support us*”. Even allowing for a false and front-stage reverence here, such statements illustrate an inherent sense of social power (cf. Scott, 1985). In Kerala, these leadership accounts were less salient within the community form. Indeed, Kerala’s earliest community organizations in Manjeri, Nilambur and Kozhikode explicitly espoused guidelines that “All are equal”, “There is no leader”, “Every volunteer is a leader” ( Rajagopal & Kumar, 1999), inscribing these guidelines into organizations’ by-laws, and training manuals ( Vijay, 2012).

***Teams and team leader.*** Marking a discursive variation in the transfer of palliative care from Kerala to Nadia, the term “community” itself was almost absent in our participants’ narratives. Rather the term “team” was used repeatedly. The discourse of community is more entrenched in Kerala, which has a history of community mobilization programmes, especially in healthcare ( Jeffrey, 2011). In contrast, the more ubiquitous and familiar concept of “team” prevailed in Nadia. Consistent with the team metaphor, the team leader’s role at Sanjeevani was to motivate, mobilize and co-ordinate. Consider how Mahesh*, a unit coordinator, describes organizing at the unit level:

When I hear that there is a patient, within 2–5 minutes, our volunteers will go to that patient … Our volunteers collect the necessary information and bring it to us. We have a team leader, they’ll send them to the team leader. In 5 minutes, the message will reach us. We cover 143 villages, in every village, we have 30–40 patient volunteers.

Mahesh’s description illustrates the hierarchies in information flow and decision-making we observed. Although co-ordination is essential to any such geographically dispersed activities, community organizations typically decentralize decision-making, emphasizing horizontal linkages and deliberative participation ( Aveling *et al.*, 2017). For instance, in Kerala’s palliative care community organizations, coordinator or administrative positions may be rotated among members ( Vijay, 2012). At Sanjeevani, the village-level volunteers are crucial nodes for patients, but unit coordinators like Mahesh emerged as leader figures.

Thus, the top-down seeding of Sanjeevani by a powerful, influential coalition comprising the DM, physicians and the IPM, created distinct vertical linkages with associated vocabularies of team and team leader.

### Translating social participation: Occupational prestige and camaraderie

How did social participation cohere amidst considerable material difficulties and top-down seeding of community organizing? Our findings suggest that newfound occupational prestige and camaraderie mobilized and constituted social participation. Sanjeevani’s volunteers came from diverse backgrounds including retired school teachers, home-makers, young unemployed graduates, and activists, with a greater number of female volunteers. Several volunteers were from economically-deprived backgrounds. Sharmishta ^*^, a volunteer shared her situation:

We come from a BPL [Below Poverty Line] family. My husband does odd jobs at people's houses. We have personal problems at home, but I love this work so I continue to do it. My husband never prevents me from working. In fact, I used to leave my baby at home. I perform all household duties, take care of my baby and then come here. Most of us do this. But I want to do this, because I love the job.

Sharmishta was unemployed and had attended the Sanjeevani training sessions with her infant. Since the 1990s, West Bengal has faced fractured industrial growth and increased unemployment. Consequently, like Sharmishta and her husband, many are compelled to seek employment in the informal sector, with commensurately lower wages ( Sen, 2009).

***Occupational prestige.*** Our participants shared with us the respect and status they garnered through their care labour. For some female volunteers, engaging in Sanjeevani’s work meant breaching the domestic sphere, and garnering respect from their families for the care they gave. Consider Moumita*: “ *We feel good because despite being housewives, we can go out and stand beside people who need support.”* Moumita reveals how women, otherwise confined to the domestic sphere, can now engage in the public sphere.

A distinctive feature at Sanjeevani was the practice of designing and wearing uniforms adopted by the volunteers and nurses. During our visit in October 2015, we were intrigued by the cream front-and-back aprons and sashes that volunteers were wearing with *Sanjeevani Palliative Care Society* imprinted. By November 2016, the nurses had a distinct dark blue salwar kameez with a white coat that distinguished them from the volunteers. Papia ^*^, a nurse, describes this sartorial change:

Earlier when we used to wear the apron while visiting patients, people did not give us much importance. But now, when we go in a group in this uniform, people approach us. This gives us motivation to work.

Uniforms are important status markers in the workplace, delineating occupational boundaries and playing a role in the construction of professional identities ( Rafaelli & Pratt, 1993; Timmons & East, 2011). Papia had undertaken a six-month vocational training in nursing along with her Bachelors and Masters degrees. With her new volunteer work as a nurse with Sanjeevani, Papia finds the uniform a means to create an occupational hierarchy. The cream apron that both volunteers and nurses wore is no longer good enough; the uniforms give them importance. Ghazal ^*^, a volunteer nurse, who was “unemployed and sitting at home” before joining Sanjeevani, justifies the uniform as follows:

When we are going to nurse someone, we should look like a nurse. If I wear a bright red *churidar* [traditional attire] and visit a patient, it does not look nice. Our uniform gives us an identity. Many people do not know about Sanjeevani, but when they see us in this blue uniform, they ask us about palliative care, they understand about Sanjeevani. This helps us to spread our work among the people.

The uniform here is an emblematic visible symbol by which a lay person can identify an occupational boundary, increasing the prestige of the nurse’s work ( Timmons & East, 2011). Ghazal is not paid for her work, but she states that she derives meaning from her engagement with patients. The uniform produces a ‘stratified homogeneity’, distinguishing the nurses from other volunteers and indicating status and hierarchy, and producing a collective identity within the group ( Rafaelli & Pratt, 1993). Worth consideration is that the uniform is coloured blue and white - the colour code of West Bengal’s ruling party, the Trinamool Congress. Mamata Banerjee, Chief Minister of West Bengal, and head of the Trinamool Congress, is quintessentially clad in white sarees with blue borders, and has been known to endorse a uniform colour code, painting government buildings, footpaths, flyovers, taxis, bus stops and tree trunks in blue and white ( Rana, 2012). In a ‘party society’ that survives on political patronage, the hidden transcripts of the nurses’ blue and white uniforms seem to have a deeper significance.

***Camaraderie.*** Volunteers also reported a shared sense of camaraderie with other volunteers and the excitement of getting out of their homes for a good cause. Mirroring a common narrative of how volunteers found it meaningful to spend time with patients, Suja*, a nurse and unit coordinator, explained:

There are patients whose families do not care for them. When we go and talk to them, they feel very happy … For them, one week's wait was a long time. They share their thoughts and feelings with us, not with their family members. They tell us about their pains and griefs and we try to console them. They say that they don’t need medicines. They just want us to come and chat with them. This is very motivating.

This form of social support provided multiple benefits to patients, including access to ambulance transport, free medication for a destitute family, pain relief by the visiting nurses home care team, and moral and spiritual support from the volunteers. Volunteers spent time with abandoned, elderly patients and read out from scriptures of the patient’s religion. Volunteers enabled patients’ access to chemotherapy and dialysis provided at a subsidized cost at the district hospital, a service of which many patients were unaware. To make use of this facility, a patient’s family had to get a document from the panchayat affirming their need for a subsidy. If the family was illiterate, or hard-pressed because the breadwinner was sick, Sanjeevani volunteers helped families with panchayat procedures.

These practices which produced sociality were not just enacted between volunteers and patients, but also among volunteers. Ritu* with 9 years of prior nursing experience shared:

I wanted to work there; I did not want money. I just liked the job. I am that kind of a person, who wants to love everyone. Elderly people − I like to talk to them. Listening to them talk about their griefs would wet my eyes. I could empathise with them. When I visit the patients, I talk a lot with them. I make them laugh when they are sad. All these people are there with me. They also laugh at my jokes and that comforts the patients.

Ritu* describes a collective production of comfort and conviviality at the site of caregiving. She elaborates,

I love this work so much that when I am at home, I feel very bored. Whenever I am on duty, and I visit patients, all my boredom vanishes and I am really happy. More than 12 hours a day I stay busy with this work. I rarely stay at home. Even after my duty is over, we stay back, chat with each other for an hour and then go home.

Ghazal* agrees: “ *We share a strong bond of friendship. Most of us are students. So, there is an energy in us … Gossiping came very naturally to me. Sometimes even the patients' parents sit with us and participate in our stories and gossips. That is how our work is spreading.*” Sanjeevani volunteers expanded these affective spaces beyond palliative care provision through various ceremonies and symbolic rituals, such as an annual picnic. Volunteers explained how they helped each other out if there were personal difficulties at home such as an illness or tragedy (like a house fire). On World Hospice and Palliative Care Day, volunteers identified new palliative patients and took sweets to patients’ homes. Intriguingly, on Raksha Bandhan, a festival in India where sisters tie a *rakhee* (a wrist band) on their brothers with the vow that the brothers will look after them, Sanjeevani volunteers took *rakhees* for patients to tie on the volunteers’ wrists. Volunteers partnered with local social work organizations (e.g., the Lions Club) to distribute wheelchairs to those in need, and organized blood donation camps. For Christmas, volunteers organized camps at a fair where, in addition to spreading awareness about palliative care, they served as a nodal point for diverse activities like first aid and keeping track of lost children.

### Impact

Sanjeevani commenced in September 2014 in Krishnanagar municipality and seven adjoining panchayats. By November 2016, Sanjeevani functioned with approximately 900 volunteers through eight units encompassing 30 panchayats. Each unit had a coordinator selected by the core committee. Consistent with Kerala’s community form, Sanjeevani’s participation evolved with volunteers becoming the locus of care-giving and decision-making. Local volunteers surveyed neighbourhoods for patients, identified needs, mapped home-care schedules, maintained regular contact with families, and provided updates to home care teams. Sanjeevani held participatory monthly review meetings and general meetings where volunteers deliberated on organizational problems, such as challenges in identifying patients, recruitment of new volunteers, or difficulties with getting a nurse for their unit.

Two points of departure from the Kerala model surfaced. First, from the earliest days in Kerala, actors articulated that “all members are equal”, “there is no leader”, or “all volunteers are leaders” ( Rajagopal & Kumar, 1999; Vijay, 2012). Indeed, the first community organization in Kerala at Nilambur, continues to run with predominantly non-medical professionals like farmers, teachers, bus-drivers and so on. Nadia, by contrast, was seeded by a distinct leader figure. Moreover, physicians were not just necessary professional support, but also pivotal to Sanjeevani’s day-to-day operations. Second, the Kerala model thrives on micro-funding often of as little as Rupees 10 (21 US cents), raised from local subaltern actors ( Quality of Death Report, 2010). Volunteers in Kerala emphasize the need for community-based micro-funding, to create community ownership ( Vijay, 2012). Given the degree of destitution, Sanjeevani members reported difficulties in local fund-raising and primary funding came from physicians, local politicians and philanthropists. Table 2 outlines key distinctions between the Kerala community form and Sanjeevani.

**Table 2.** Translation of Kerala’s community form to Nadia.

	Kerala community form	Sanjeevani community form
Member relations	Community ownership	Community participation: combination of hierarchies and horizontal linkages
Organizational decision-making	Deliberative, horizontal	Primarily hierarchical – decisions run through physicians, unit coordinators team leaders
Practices - Caregiving	Nurses and volunteer home-care, physician’s home care, physiotherapists	Nurses and volunteer home care, physician’s home care, physiotherapists
Practices - Fund-raising	Micro-funds from neighborhoods	Big donors – e.g. physicians, politicians.
Operationalizing total care	Social, emotional, spiritual, financial, Medical, bereavement Support, rehabilitation	Social, emotional, spiritual, medical, bereavement support
Patient categories	Cancer, HIV/AIDS, Chronic renal, respiratory, cardiovascular conditions, Geriatric conditions, Paraplegia, Mental Health	Advanced Cancer, Bedridden patients, Geriatric conditions (over 75 years).
Ties with state actors	Formal integration into public health system	Patronage/ donor-based relations with local politicians

Sources: Primary data sources; Kumar, 2013; Vijay, 2012; Vijay & Kulkarni, 2012

## Discussion

In this research, we analyzed how the dynamics of translation influenced the transfer of a community form for palliative care to Nadia, in West Bengal. We now explore three key contributions from our study. First, our research extends contemporary discussions which suggest a shift from an analytic focus on *transfer* of healthcare interventions from a dominant context to peripheral contexts, and towards *translation* processes (e.g. Kothari & Armstrong, 2011; Lawrence, 2017; Mannell, 2014; Montesanti *et al.*, 2017). This translation lens allows us to anticipate plural futures for palliative care, beyond the dominant hospice and hospital-based forms (cf. Zaman *et al.*, 2017). We foreground institutional analysis in our study of the translation process.

The unfettered real-time data access that we encountered in Nadia presented an excellent opportunity for an enquiry into translation as an ongoing, dynamic process. The symbolic capital of the imported practice (i.e., what is translated) and global legitimacy of the source context (i.e., from where) are significant during translation ( Gouanvic, 2010). However, Kerala’s community intervention had been confined primarily within state boundaries, and prior to the training program, most participants at Nadia were unaware of palliative care, or of the Kerala model. Our narrative highlights the processual elements, as actors interpret a health service delivery model developed in another institutional context, encounter local resistance and material challenges and constitute community participation through salient values (here, occupational prestige and camaraderie). In this way, across the study period, we observed over 900 community volunteers mobilize for ‘total care’ of patients in need of palliative care.

In healthcare translations, we attend to organizations’ strategic decisions to engage communities (e.g. Mannell, 2014; Montesanti *et al.*, 2015), particularly by powerful actors such as policy makers, program directors, and managers, without necessarily attending to the ongoing processes by which participation coheres among members at the frontline. This perhaps arises out of a methodological constraint, where “ *in vivo* and *in situ* studies of editors or translators based on direct observation in real time” are rare ( Zilber, 2006, p. 300). Our processual account delineates how non-elites interpret, give meaning to, transform and adopt the received intervention, and the affective dimensions of this engagement.

Stark economic deprivation and weak institutional histories of civil society organization present organizational challenges. Accordingly, translator’s employment of symbolic power, and vertical solidarities forged among local elites with community members are key mechanisms for resource mobilization and overcoming low organizational legitimacy. Further, in this non-remunerative context, occupational prestige and camaraderie serve as affective mechanisms of institutional work that serve to cohere social participation. Given Nadia’s institutional context, the translated community form materialized as a hybrid form, with vertical and horizontal relational structures. Community interventions, such as the Kerala model, are typified as non-hierarchical settings with bottom-up consensus building ( Aveling *et al.*, 2012; Santhosh, 2016; Vijay, 2012). At Nadia, a hybrid community form spearheaded by leaders, may be a contextually appropriate and necessary variation for implementation.

Indeed, Aveling *et al*. (2017) point to the merit of ‘clinical communities’ that have a blend of a vertical core and horizontal peer linkages. Unlike ‘clinical communities’, Sanjeevani does not have pre-figured structures and activities prior to the translation process. Rather, certain actors, like Mahesh and Suja, emerge as leaders from the community during translation. Nevertheless, like clinical communities, formulating the Nadia form as one such hybrid of vertical and horizontal linkages, can serve as a prototype for community palliative care interventions in contexts that do not have similar histories of bottom-up organizing like Kerala.

Second, healthcare transfer policies emphasize rational and strategic aspects of evidence-based medical decision-making and implementation frameworks to facilitate desirable health outcomes, resource mobilization, coalition formation, and so on ( Kothari & Armstrong, 2011; Montesanti *et al.*, 2017). Foregrounding these rational, cognitive aspects of social life obscures affective elements so integral to caregiving. Our findings point to how, despite the absence of economic incentives, participation is forged around the occupational prestige and camaraderie that members derive. Indeed, research on affects, which draws attention to body, emotions, feelings and passions, delineates how affective labor is productive of socialities, from households and families, to communities and movements ( Ahmed, 2004; Hardt, 1999). Future research could shed more light on how affects of prestige, status, camaraderie and conviviality serve as the glue in community-based action in subaltern settings.

Third, we argue that the concept of community is operationalized uncritically in healthcare policies without acknowledging its contested nature ( Bertotti *et al.*, 2012; Crow & Allan, 1994; Montesanti *et al.*, 2015; Montesanti *et al.*, 2017). Studies presume communities as already constituted, with shared identities and connectedness, poised to serve as low-cost, resource-effective vehicles for participatory interventions ( Lippman *et al.*, 2018; Mansuri & Rao, 2004). We illustrate that ‘community’ cannot be taken as a pre-existing social category in the target field and careful attention must be given to how community participation is mobilized and constituted during translation. Moreover, if organizations are birthed in the institutional legacies of communities ( Greve & Rao, 2014), it follows that community forms like Sanjeevani can result in better contextual readiness for future community health interventions.

In sum, our work cautions against simplistic, decontextualized transfer models in global health discourse, and especially those related to palliative care ( World Health Organization, 2014). There have been reservations about the transfer of the Kerala model to other geographies, given its unique institutional context ( Sallnow *et al.*, 2010). Indeed, our findings illustrate how transferring a community intervention presents challenges not only across countries but also within India. Our study points to the importance of prospectively analyzing institutional contexts, during healthcare transfer experiments. Such an analysis may help identify important actors like state officials, who in such contexts, may enjoy far greater legitimacy than non-governmental actors.

There is much emphasis on the promise of communities in the 21 ^st^ century ( Ahmed & Fortier, 2003). There is also much optimism about the potential of ‘compassionate communities’ to promote new approaches to palliative care ( Zaman *et al.*, 2018). We hope our study, by illustrating the challenges and efforts of establishing and maintaining communities, contributes to a meaningful realization of the power contained within them.

## Data availability

Participant information in the primary data sources that support our findings cannot be sufficiently anonymized and de-identified. Due to these confidentiality and security considerations, the interview data cannot be shared in the public domain. Excerpts from primary data can be made available after participant information is de-identified. For access to the primary data set, please email: devivijay@iimcal.ac.in

## Supplementary material

Supplementary File 1: The 116 articles that comprised the secondary dataset.

## References

[ref-1] Abel J Walter T Carey LB: Circles of care: Should community development redefine the practice of palliative care? *BMJ Support Palliat Care.* 2013;3(4):383–8. 10.1136/bmjspcare-2012-000359 24950517

[ref-3] Ahmed S: Affective economies. *Social Text.* 2004;22(2):117–139. 10.1215/01642472-22-2_79-117

[ref-2] Ahmed S Fortier AM: Re-imagining communities. *Int J CULTURAL STUD.* 2003;6(3):251–259. 10.1177/13678779030063001

[ref-4] Aveling EL Martin G Armstrong N: Quality improvement through clinical communities: eight lessons for practice. *J Health Organ Manag.* 2012;26(2):158–174. 10.1108/14777261211230754 22856174

[ref-5] Aveling EL Martin G Herbert G: Optimising the community-based approach to healthcare improvement: Comparative case studies of the clinical community model in practice. *Soc Sci Med.* 2017;173:96–103. 10.1016/j.socscimed.2016.11.026 27936423 PMC5240788

[ref-6] Bandyopadhyay D: Is the institution of District Magistrate still necessary? *Econ Polit Wkly.* 2006;41(47):4851–4853. Reference Source

[ref-7] Bardach AE Elorriaga N Alcaraz AO: Community-based cardiovascular health promotion in Argentina. A systematic review. *Health Promot Int.* 2017; pii: daw107. 10.1093/heapro/daw107 28137729

[ref-8] Bertotti M Adams-Eaton F Sheridan K: Key Barriers to Community Cohesion: Views from Residents of 20 London Deprived Neighbourhoods. *GeoJournal.* 2012;77(2):223–234. 10.1007/s10708-009-9326-1 27761059 PMC5066839

[ref-9] Bhattacharya D: Of control and factions: The changing 'Party-Society' in rural West Bengal. *Econ Polit Wkly.* 2009;44(9):59–69. Reference Source

[ref-10] Bourdieu P Wacquant L: Symbolic capital and social classes. *Journal of Classical Sociology.* 2013;13(2):292–302. 10.1177/1468795X12468736

[ref-11] Campbell C Cornish F: How can community health programmes build enabling environments for transformative communication? Experiences from India and South Africa. *AIDS Behav.* 2012;16(4):847–857. 10.1007/s10461-011-9966-2 21604108

[ref-12] Census: Census of India. New Delhi: Government of India,2011 Reference Source

[ref-13] Choudhury M Nath HA: An estimate of public expenditure on health in India. New Delhi: National Institute of Public Finance and Policy,2012;1–17. Reference Source

[ref-14] Crow G Allen G: Community life: An Introduction to Local Social Relations. New York: Harvester-Wheatsheaf,1994 Reference Source

[ref-15] Czarniawska B Joerges B: Travels of ideas. In Czarniawska B, Sevón G. (Eds.). *Translating Organizational Change.*de Gruyter, Berlin,1996;13–48.

[ref-17] Draper AK Hewitt G Rifkin S: Chasing the dragon: Developing indicators for the assessment of community participation in health programmes. *Soc Sci Med.* 2010;71(6):1102–1109. 10.1016/j.socscimed.2010.05.016 20621405

[ref-18] Erlandson DA Harris EL Skipper BL: Doing Naturalistic Inquiry: A Guide to Methods. London: Sage.1993 Reference Source

[ref-19] Garg CC Karan AK: Reducing out-of-pocket expenditures to reduce poverty: A disaggregated analysis at rural-urban and state level in India. *Health Policy Plan.* 2008;24(2):116–128. 10.1093/heapol/czn046 19095685

[ref-20] Gouanvic JM: Outline of a sociology of translation informed by the ideas of Pierre Bourdieu. *Monografías de Traducción e Interpretación.* 2010;2:119–129. 10.6035/MonTI.2010.2.6

[ref-21] Graham F Clark D: Definition and evaluation: Developing the debate on community participation in palliative care. *Indian J Palliat Care.* 2005;11(1):2–5. 10.4103/0973-1075.16636

[ref-22] Greenwood R Suddaby R: Institutional entrepreneurship in mature fields: The big five accounting firms. *Acad Manage J.* 2006;49(1):27–48. 10.5465/amj.2006.20785498

[ref-23] Greve HR Rao H: Echoes of the past: Organizational foundings as sources of an institutional legacy of mutualism. *Am J Sociol.* 2012;118(3):635–675. 10.1086/667721

[ref-24] Greve HR Rao H: History and the present: Institutional legacies in communities of organizations. *Res Organ Behav.* 2014;34:27–41. 10.1016/j.riob.2014.09.002

[ref-25] Hardt M: Value and affect. *boundary 2.* 1999;26(2):89–100. Reference Source

[ref-26] Hardy C: Underorganized interorganizational domains: The case of refugee systems. *J Appl Behav Sci.* 1994;30(3):278–296. 10.1177/0021886394303002

[ref-27] Hwang H Suarez D: Lost and found in the translation of strategic plans and websites. In Czarniawska B, Sevon G. (Eds.), *Global Ideas: How Ideas, Objects and Practices Travel in the Global Economy.*Malmo, Sweden: Liber AG,2005;71–93. Reference Source

[ref-28] India Human Development Report, 2011. Towards Social Inclusion. Institute of Applied Manpower Research. Planning Commission, Government of India,2011 Reference Source

[ref-29] Jeffrey R: Politics, Women and Well-being: How Kerala became a Model. Oxford: Oxford University Press.2011.

[ref-30] Kothari A Armstrong R: Community-based knowledge translation: unexplored opportunities. *Implement Sci.* 2011;6:59. 10.1186/1748-5908-6-59 21645381 PMC3127775

[ref-31] Kumar S: Models of delivering palliative and end-of-life care in India. *Curr Opin Support Palliat Care.* 2013;7(2):216–222. 10.1097/SPC.0b013e3283610255 23635881

[ref-66] Lawrence TB: High-Stakes Institutional Translation: Establishing North America’s First Government-sanctioned Supervised Injection Site. *Acad Manage J.* 2017;60(5):1771–1800. 10.5465/amj.2015.0714

[ref-32] Lippman SA Leslie HH Neilands TB: Context matters: Community social cohesion and health behaviors in two South African areas. *Health Place.* 2018;50:98–104. 10.1016/j.healthplace.2017.12.009 29414427 PMC5962353

[ref-33] Mannell J: Adopting, manipulating, transforming: tactics used by gender practitioners in South African NGOs to translate international gender policies into local practice. *Health Place.* 2014;30:4–12. 10.1016/j.healthplace.2014.07.010 25151498

[ref-34] Mansuri G Rao V: Community-based and -driven development: A critical review. *World Bank Res Obs.* 2004;19(1):1–39. 10.1093/wbro/lkh012

[ref-16] Margaret Dolcini M Gandelman AA Vogan SA: Translating HIV interventions into practice: Community-based organizations’ experiences with the diffusion of effective behavioral interventions (DEBIs). *Soc Sci Med.* 2010;71(10):1839–1846. 10.1016/j.socscimed.2010.08.011 20926169 PMC2994322

[ref-35] Marquis C Lounsbury M Greenwood R: Introduction: Community as an institutional order and a type of organizing. In C. Marquis, M. Lounsbury, & R. Greenwood (Eds.), *Community and Organizations.* 2011;33:ix–xxvii. 10.1108/S0733-558X(2011)0000033003

[ref-36] McDermott E Selman L Wright M: Hospice and palliative care development in India: a multimethod review of services and experiences. *J Pain Symptom Manage.* 2008;35(6):583–593. 10.1016/j.jpainsymman.2007.07.012 18395401

[ref-37] Montesanti SR Abelson J Lavis JN: The value of frameworks as knowledge translation mechanisms to guide community participation practice in Ontario CHCs. *Soc Sci Med.* 2015;142:223–231. 10.1016/j.socscimed.2015.08.024 26318211

[ref-38] Montesanti SR Abelson J Lavis JN: Enabling the participation of marginalized populations: case studies from a health service organization in Ontario, Canada. *Health Promot Int.* 2017;32(4):636–649. 10.1093/heapro/dav118 26802073

[ref-39] Pratichi Report: Non-communicable Diseases: A Preview from West Bengal, 2012. Pratichi Institute, Kolkata.2012 Reference Source

[ref-40] Quality of Death Report: Quality of death: Ranking End-of-life Care Across the World. Economist Intelligence Unit, Londres, RU.2010 Reference Source

[ref-41] Quality of Death Report: Quality of death index: Ranking Palliative care across the World. Economist Intelligence Unit, Londres, RU.2015 Reference Source

[ref-42] Rafaelli A Pratt MG: Tailored meanings: On the meaning and impact of organizational dress. *Acad Manage Rev.* 1993;18(1):32–55. 10.2307/258822

[ref-43] Rajagopal MR: The current status of palliative care in India. *Cancer Control.* 2015;57–62. Reference Source

[ref-44] Rajagopal MR Kumar S: A model for delivery of palliative care in India--the Calicut experiment. *J Palliat Care.* 1999;15(1):44–9. 10333664

[ref-45] Rana P: Kolkata’s Blue Makeover Has Some Seeing Red.2012; Accessed on 10 ^th^June 2016. Reference Source

[ref-46] Rifkin S: Health planning and community participation. *World Health Forum.* 1986;7(2):156–162. Reference Source

[ref-47] Roy D: Caste and power: An ethnography in West Bengal, India. *Mod Asian Stud.* 2012;46(4):947–974. 10.1017/S0026749X11000680

[ref-48] Sahlin K Wedlin L: Circulating ideas: Imitation, translation and editing. In Greenwood, R., Oliver, C., Suddaby, R. Sahlin-Andersson, K. (Eds). *The Sage Handbook of Organizational Institutionalism.*London: Sage,2008;218–242. 10.4135/9781849200387.n9

[ref-50] Sallnow L Kumar S Kellehear A: International Perspectives on Public Health and Palliative Care. Abingdon: Routledge.2012 Reference Source

[ref-49] Sallnow L Kumar S Numpeli M: Home-based palliative care in Kerala, India: The Neighborhood Network in Palliative Care. *Prog Palliat Care.* 2010;181:14–17. 10.1179/096992610X12624290276142

[ref-51] Santhosh R: Voluntarism and civil society in the neoliberal era: A study on the palliative care movement in Kerala. *J Soc Econ Develop.* 2016;18(1–2):1–16. 10.1007/s40847-016-0024-9

[ref-52] Scott JC: Weapons of the Weak. Everyday Forms of Peasant Resistance.New Haven: Yale University Press,1985 Reference Source

[ref-53] Sen R: The Evolution of Industrial Relations in West Bengal. Geneva: International Labor Organization.2009 Reference Source

[ref-67] Strauss A Corbin JM: Basics of qualitative research: Grounded theory procedures and techniques. Thousand Oaks, CA, US: Sage Publications, Inc.,1990 Reference Source

[ref-54] Timmons S East L: Uniforms, status and professional boundaries in hospital. *Sociol Health Illn.* 2011;33(7):1035–1049. 10.1111/j.1467-9566.2011.01357.x 21507013

[ref-68] Tracey P Dalpiaz E Phillips N: Fish out of Water: Translation, Legitimation, and New Venture Creation. *Acad Manage J.* 2018 10.5465/amj.2015.0264

[ref-55] Vijay D: Collective Action Frame and Organizational Field Emergence in The Context of Palliative Care in Kerala, India. Unpublished Dissertation.2012.

[ref-57] Vijay D Ghosh D: The Sabar Shouchagar Project (Toilets for everyone): Making Nadia District the first open-defecation-free district in India. *Emerald Emerging Markets Case Studies.* 2018;8(1):1–25. 10.1108/EEMCS-03-2017-0061

[ref-56] Vijay D Kulkarni M: Frame changes in social movements: a case study. *Public Manag Rev.* 2012;14(6):747–770. 10.1080/14719037.2011.642630

[ref-58] Wolf M Fukari A (Eds.): Constructing a Sociology of Translation. John Benjamins Publishing, Philadelphia.2007;74 Reference Source

[ref-59] World Bank Group.2012 Reference Source

[ref-60] World Health Organization: Declaration of Alma-Ata. *International Conference on Primary Health Care, 6–12 September, Alma-Ata, USSR.*Geneva, World Health Organization.1978 Reference Source

[ref-61] World Health Organization: Strengthening of Palliative care as a component of comprehensive care throughout the life course.2014; Accessed on 6th November 2017. Reference Source

[ref-62] World Health Organization: WHO definition of palliative care.2017; Accessed on 6 ^th^November 2017. Reference Source

[ref-63] Zaman S Inbadas H Whitelaw A: Common or multiple futures for end of life care around the world? Ideas from the ‘waiting room of history’. *Soc Sci Med.* 2017;172:72–79. 10.1016/j.socscimed.2016.11.012 27894008 PMC5224187

[ref-64] Zaman S Whitelaw A Richards N: A moment for compassion: emerging rhetorics in end-of-life care. *Med Humanit.* 2018; pii: medhum-2017-011329. 10.1136/medhum-2017-011329 29440385 PMC6031266

[ref-65] Zilber TB: The work of the symbolic in institutional processes: Translations of rational myths in Israeli high tech. *Acad Manage J.* 2006;49(2):281–303. 10.5465/AMJ.2006.20786073

