# Supplementary material for: Translation of a community palliative care intervention: Experience from West Bengal, India
Source: Wellcome Open Res. 2018 May 31;3:66. [Version 1] doi: 10.12688/wellcomeopenres.14599.1 (PMC6069742; doi:10.12688/wellcomeopenres.14599.1)
Supplement: Supplementary file 1 [file wellcomeopenres-3-15895-s0000.tgz › 75984ac2-db21-4611-a6e3-94ccb212ea15.docx]

**BIBLIOGRAPHY**

1. Abel, J., Walter, T., Carey, L. B., Rosenberg, J., Noonan, K., Horsfall, D., Leonard, R., Rumbold, B., & Morris, D. (2013). Circles of care: Should community development redefine the practice of palliative care? *BMJ Supportive & Palliative Care*, 3, 383-388.
2. Abraham, A.A. (2011). *Impact of Community-Owned Home-Based Palliative Care on Quality of Life of Cancer Patients in Kerala.* Doctoral dissertation, SCTIMST. Trivandrum, Kerala.
3. Ajithakumari, K., Kumar, S. K., & Rajagopal, M.R. (1997). Palliative home care— The Calicut experience. *Palliative medicine*, *11*(6), 451-454.
4. Bollini, P., Venkateswaran, C., & Kumar, S.K. (2004). Palliative care in Kerala, India: A model for resource-poor settings. *Oncology Research and Treatment*, *27*(2), 138-142.
5. Brennan, F., Gwyther, L., & Harding, R. (2006). *Palliative Care as a Human Right*. New York, USA: Open Society Institute Public Health Program.
6. Burn, G. (1990). A personal initiative to improve palliative care in India. *Palliative Medicine*, *4*(4), 257-259.
7. Burn, G. (1997). From paper to practice: Quality of life in a developing country. *Annals of the New York Academy of Sciences*, *809*(1), 249-260.
8. Burn, G. (2001). A personal initiative to improve palliative care in India: 10 years on. *Palliative medicine*, *15*(2), 159-162.
9. Calicut Declaration (1997). Calicut Declaration. *Indian Journal of Medical Ethics.* <http://www.issuesinmedicalethics.org/061re028.html> Accessed on 1^st^ December 2010
10. Chatterjee, S. C., & Sengupta, J. (2017). *Death and Dying in India: Ageing and End-of-life Care of the Elderly*. New York: Routledge Contemporary South Asia.
11. Chaturvedi, S. K., & Chandra, P. S. (1998). Palliative care in India. *Supportive Care in Cancer*, *6*(2), 81-84.
12. Chaturvedi, S. K. (1991). What's important for quality of life to Indians—in relation to cancer. *Social Science & Medicine*, *33*(1), 91-94.
13. Chaturvedi, S. (2003). What's important for quality of life to Indians in relation to cancer. *Indian Journal of Palliative Care*, *9*(2), 62-70.
14. Chaturvedi, S. K. (2008). Ethical dilemmas in palliative care in traditional developing societies, with special reference to the Indian setting. *Journal of medical ethics*, *34*(8), 611-615.
15. Chittazhathu, R., & Moideen, S. (2005). Training community volunteers and professionals in the psychosocial aspects of palliative care. *Indian Journal of Palliative Care*, *11*(1), 53-54.
16. Clark, D. (2007). From margins to centre: A review of the history of palliative care in cancer. *The Lancet Oncology*, *8*(5), 430-438.
17. Clemens, K. E., Kumar, S., Bruera, E., Klaschik, E., Jaspers, B., & De Lima, L. (2007). Palliative care in developing countries: what are the important issues? *Palliative Medicine*, *21*(3), 173-175.
18. Declaration by the People of Kerala (2015). <https://palliumindia.org/cms/wp-content/uploads/2015/02/Declaration-by-the-People-of-Kerala-2-Feb-2015.pdf> Accessed on 1^st^ March 2018.
19. Emanuel, N., Simon, M.A., Burt, M., Joseph, A., Sreekumar, N., Kundu, T., Khemka, V., Biswas, B., Rajagopal, M.R., Emanuel, L. (2010). [Economic impact of terminal illness and the willingness to change it.](http://www.ncbi.nlm.nih.gov/pubmed/20712463) ***Journal of Palliative Medicine*** 13(8), 941-944
20. Fox, H., & Jackson, K. (2015). Pain, palliative care, and compassion in India. *Journal of Pain & Palliative Care Pharmacotherapy*, *29*(4), 412-415.
21. Government of Kerala. (1999). *Narcotics and Psychotropic Substances Act*. <https://palliumindia.org/cms/wp-content/uploads/2014/10/KERALA-NDPS-rules-Gazette-notification.pdf>
22. Government of Kerala. (2008). *Pain and Palliative Care Policy for Kerala*. Trivandrum, Kerala.
23. Government of Kerala – Arogyakeralam Report. (2008). *Arogyakeralam Project in Palliative Care – An update*. Trivandrum, Kerala.
24. Government of Kerala. (2009). *Implementation of Pain and Palliative Care Policy.* Circular. PH 6/068463, 02 July 2009. Directorate of Health Services.
25. Graham, F., & Clark, D. (2005). Definition and evaluation: Developing the debate on community participation in palliative care. *Indian Journal of Palliative Care*, *11*(1), 2-5.
26. Gupta, H. (2005). Community participation in palliative care: A comment. *Indian Journal of Palliative Care*, *11*(1), 19-21.
27. Harris, J. T., Kumar, K. S., & Rajagopal, M. R. (2003). Intravenous morphine for rapid control of severe cancer pain. *Palliative Medicine*, *17*(3), 248-256.
28. HelpAge India (2011). *A Report on Evaluation of Community Managed Palliative Care Plus Project*. Helpage India Report.
29. Inbadas, H., Zaman, S., Whitelaw, A., & Clark, D. (2016). Palliative care declarations: Mapping a new form of intervention. *Journal of Pain and Symptom Management*, *52*(3), e7-e15.
30. Indian Association of Palliative Care & CanSupport. (2006). *Guidelines for Home-Based Palliative Care Services*. New Delhi, India
31. Institute of Palliative Medicine (2017). *Palliative Care – A Workbook for Carers*. Puducherry, India.
32. Jack, B. A., Kirton, J., Birakurataki, J., & Merriman, A. (2011). ‘A bridge to the hospice’: The impact of a community volunteer programme in Uganda. *Palliative medicine*, *25*(7), 706-715.
33. Jack, B. A., Kirton, J. A., Birakurataki, J., & Merriman, A. (2012). The personal value of being a palliative care community volunteer worker in Uganda: a qualitative study. *Palliative medicine*, 26(5), 753-759.
34. Jayalakshmi, R., & Suhita, C. C. (2017). Home-based palliative services under two local self-government institutions of Kerala, India: An assessment of compliance with policy and guidelines to local self-government institutions. *Indian Journal of Palliative Care*, *23*(1), 65-70.
35. Jayalakshmi R, Chatterjee SC, Chatterjee D. (2016). End-of‑life characteristics of the elderly: An assessment of home‑based palliative services in two panchayats of Kerala. *Indian Journal of Palliative Care*. 22, 491‑498.
36. Joranson, D. E., Rajagopal, M. R., & Gilson, A. M. (2002). Improving access to opioid analgesics for palliative care in India. *Journal of Pain and Symptom Management*, *24*(2), 152-159.
37. Joseph, N., Jayarama, S., & Kotian, S. (2009). A comparative study to assess the awareness of palliative care between urban and rural areas of Ernakulum district, Kerala, India. *Indian Journal of Palliative Care*, *15*(2), 122-126.
38. Kerala State Human Rights Commission (2014). Kerala State Human Rights Commission G.O.

<https://palliumindia.org/cms/wp-content/uploads/2015/02/KSHRC-order-dt-30-10-2014-translated.pdf> Accessed 1^st^ March 2018

1. Khosla, D., Patel, F. D., & Sharma, S. C. (2012). Palliative care in India: current progress and future needs. *Indian Journal of Palliative Care*, *18*(3), 149-154.
2. Knaul, F.M., Farmer, P.E., Krakauer, E.L., De Lima, L., Bhadelia, A., Jiang Kwete, X., Arreola-Ornelas, H., Gómez-Dantés, O., Rodriguez, N.M., Alleyne, G.A.O., Connor, S.R., Hunter, D.J., Lohman, D., Radbruch, L., Del Rocío, M., Atun, R., Foley, K.M., Frenk, J., Jamison, D.T., Rajagopal, M.R. (2017). Alleviating the access abyss in palliative care and pain relief— an imperative of universal health coverage: The Lancet Commission report. *The Lancet*. 391, 1391-1454
3. Korankulangara, T. *Enabling Factors in Neighborhood Network in Palliative Care (NNPC): An Exploratory Study in Kozhikode District, Kerala*. Master’s Thesis
4. Koshy, C. (2009). The palliative care movement in India: Another freedom struggle or a silent revolution?. *Indian Journal of Palliative Care*, *15*(1), 10-13.
5. Krakauer, E.L., Rajagopal, M.R. (2016). End-of-life care across the world: a global moral failing. *Lancet*. 388, 444-6.
6. Kumar, A. (2004). Organization and development of pain clinics and palliative care in developing countries. *European Journal of Anaesthesiology,* 21, 169-172.
7. Kumar, S. (2004). Learning from low income countries: what are the lessons?: Palliative care can be delivered through neighbourhood networks. *BMJ: British Medical Journal*, *329*(7475), 1184.
8. Kumar, K. S., & Rajagopal, M. R. (1996). Problems at presentation in 440 patients with advanced cancer in a south Indian state. *Palliative Medicine*, *10*(4), 293-298.
9. Kumar, K. S., Rajagopal, M. R., & Naseema, A. M. (2000). Intravenous morphine for emergency treatment of cancer pain. *Palliative Medicine*, *14*(3), 183-188.
10. Kumar, S. (2005). Community programmes in palliative care: What have we learned?. *Indian Journal of Palliative Care*, *11*(1), 55-57.
11. Kumar, S. K. (2007). Kerala, India: A regional community-based palliative care model. *Journal of Pain and Symptom Management*, *33*(5), 623-627.
12. Lamas, D., & Rosenbaum, L. (2012). Painful inequities— Palliative care in developing countries. *New England Journal of Medicine*, *366*(3), 199-201.
13. Mathew, A., & Rajagopal, M.R. (2016). Care of the Terminally Ill Patient in India: Comments on the Proposed Legislation. *Journal of Global Oncology*. 3(3), 278-279.
14. McDermott, E., Selman, L., Wright, M., & Clark, D. (2008). Hospice and palliative care development in India: a multimethod review of services and experiences. *Journal of Pain and Symptom Management*, *35*(6), 583-593.
15. Morris, S., Wilmot, A., Hill, M., Ockenden, N., & Payne, S. (2013). A narrative literature review of the contribution of volunteers in end-of-life care services. *Palliative Medicine*, *27*(5), 428-436.
16. Namukwaya, E., Leng, M., Downing, J., & Katabira, E. (2011). Cancer pain management in resource-limited settings: A practice review. *Pain Research and Treatment*.  Doi:10.1155/2011/393404
17. Open Society Institute (2007). *Palliative Care and Human Rights - A Resource Guide*. New York, USA: Open Society Institute
18. Palat, G., & Venkateswaran, C. (2012). Progress in palliative care in India. *Progress in Palliative Care*, *20*(4), 212-218.
19. Palat, G. & Rajagopal, M. (2006). Pain relief on a shoe-string budget: Experience from Kerala, India. Proceedings of the 11th World Congress on Pain. IASP Press, Seattle.
20. Paleri, A., & Numpeli, M. (2005). The evolution of palliative care programmes in North Kerala. *Indian Journal of Palliative Care*, *11*(1), 15-18.
21. Paleri, A. (2008). Showing the way forward: Pain and Palliative Care Policy of the Government of Kerala. *Indian Journal of Palliative Care*, *14*(1), 51-54.
22. Potts, M., Cartmell, K. B., Nemeth, L., Bhattacharjee, G., & Qanungo, S. (2018). A systematic review of palliative care intervention outcomes and outcome measures in low resource countries. *Journal of Pain and Symptom Management*.
23. Powell, R.A., Blanchard, C.L., de Lima, L, Connor, S.R., Rajagopal, M.R. (2015). Cancer therapy in developing countries: The role of palliative care. In Alt-Epping, B., Nauck, F. (Eds). *Palliative Care in Oncology*. New York: Springer.
24. Quality of Death Index. (2010). The 2010 Quality of Death Index. Ranking palliative care across the world. *London: The Economist Intelligence Unit*.
25. Quality of Death Index (2015). The 2015 Quality of Death Index. Ranking palliative care across the world. *London: The Economist Intelligence Unit*.
26. Raghavan, B., Palat, G., & Rajagopal, M. R. (2005). Are our patients getting palliative care too late? An audit. *Indian Journal of Palliative Care*, *11*(2), 108.
27. Rajagopal, M. R. (2001). The challenges of palliative care in India. *National Medical Journal of India*, *14*(2), 65-67.
28. Rajagopal, M.R. (2003). From India. *Palliative Medicine*, 17,156.
29. Rajagopal, M. R. (2006). Pain and beyond. *Indian Journal of Palliative Care*, *12*(1), 4-7.
30. Rajagopal, M. R. (2007). Palliative care: an urgent need for most of the world. *International journal of environmental studies*, *64*(3), 301-307.
31. Rajagopal, M.R. (2009). Pain, suffering and pain relief – a global perspective. ***British Pain News*.** Spring 2009. 22-24.
32. Rajagopal, M. R. (2010). Disease, dignity and palliative care. *Indian journal of palliative care*, *16*(2), 59.
33. Rajagopal M.R. (2011) [Where is the evidence for pain, suffering, and relief – can narrative help fill the void?](http://www.ncbi.nlm.nih.gov/pubmed/21426214) *Journal of Pain Palliative Care Pharmacotherapy*, 25 (1):25-8.
34. Rajagopal, M. R. (2015). The current status of palliative care in India. *Cancer control*, 57-62.
35. Rajagopal, M.R. (2016). Access to palliative care: insights into ground realities post-2014 amendment to NDPS Act. *Indian Journal of Medical Ethics*. 1(1):25-30
36. Rajagopal, M.R. (2016). We have a Responsibility. *Indian Journal of Palliative Care*. 22(3), 239-43.
37. Rajagapol, M.R., Mazza, D., Lipman, A. (2004). *Pain and Palliative Care in the Developing World and Marginalized Populations: A Global Challenge*. New York, USA: Haworth Medical Press.
38. Rajagopal, M. R., Karim, S., & Booth, C. M. (2017). Oral morphine use in South India: A population-based study. *Journal of Global Oncology*, *3*(6), 720-727.
39. Rajagopal, M.R., Khan, A.J., Muckaden, M., George, R., Gupta, H., Leng, M.E.F., Palat, G., Patel, F., Raghaven, B., Reddy, S.K., Sunilkumar, M.M., Tiruvadanan, M., Connor, S.R. (2014). Creation of minimum standard tool for palliative care in India and self-evaluation of palliative care programs using it. *Indian Journal of Palliative Care*, 20, 201-207.
40. Rajagopal, M. R., & Kumar, S. (1999). A model for delivery of palliative care in India--the Calicut experiment. *Journal of Palliative Care*, *15*(1), 44.
41. Rajagopal, M.R., & George, R. (2015). Providing palliative care in economically disadvantaged countries. In Cherny, N., Fallon, M., Kaasa, S., Portenoy, R.K., Currow, D.C. (Eds.), *Oxford Textbook of Palliative Medicine.* 5th ed. Oxford: Oxford University Press.
42. Rajagopal, M. R., Joranson, D. E., & Gilson, A. M. (2001). Medical use, misues, and diversion of opioids in India. *The Lancet*, *358*(9276), 139-143.
43. Rajagopal, M. R., & Joranson, D. E. (2007). India: Opioid availability—an update. *Journal of Pain and Symptom Management*, *33*(5), 615-622.
44. Rajagopal, M. R., & Palat, G. (2002). Kerala, India: Status of cancer pain relief and palliative care. *Journal of pain and symptom management*, *24*(2), 191-193.
45. Rajagopal, M. R., & Twycross, R. (2011). Providing palliative care in resource-poor countries. *Oxford Textbook of Palliative Medicine*, *4*, 23-31.
46. Rajagopal, M.R., Vallath, N., Mathews, L., Rajashree, K.C., Watson, M. (2015). *An Indian Primer of Palliative Care*. Trivandrum, Kerala: Trivandrum Institute of Palliative Sciences
47. Rajagopal, M. R., & Venkateswaran, C. (2004). Palliative care in India: Successes and limitations. *Journal of pain & palliative care pharmacotherapy*, *17*(3-4), 121-128.
48. Ramesh, P. R., Santhosh, A. R., & Kumar, K. S. (1998). Topical morphine in Ayurveda. *Palliative medicine*, *12*(1), 64-64.
49. Ramesh, P. R., Kumar, K. S., Rajagopal, M. R., Balachandran, P., & Warrier, P. K. (1998). Managing morphine-induced constipation: a controlled comparison of an Ayurvedic formulation and senna. *Journal of Pain and Symptom Management*, *16*(4), 240-244.
50. Reynolds, L. A., & Tansey, E. M. (2004). *Innovation in Pain Management*. London, UK: Wellcome Trust Centre for the History of Medicine.
51. Sallnow, L., & Chenganakkattil, S. (2005). The role of religious, social and political groups in palliative care in Northern Kerala. *Indian Journal of Palliative Care*, *11*(1), 10-14.
52. Sallnow, L., Kumar, S., & Numpeli, M. (2010). Home-based palliative care in Kerala, India: The neighbourhood network in palliative care. *Progress in Palliative Care*, *18*(1), 14-17.
53. Sallnow, L., Kumar, S., & Kellehear, A. (Eds.). (2013). *International Perspectives on Public health and Palliative Care*. New York: Routledge.
54. Sallnow, L., Richardson, H., Murray, S. A., & Kellehear, A. (2016). The impact of a new public health approach to end-of-life care: A systematic review. *Palliative Medicine*, *30*(3), 200-211.
55. Santha, S. (2011). Impact of pain and palliative care services on patients*. Indian Journal of Palliative Care*. 17, 24-32.
56. Santhosh, R. (2016). Voluntarism and civil society in the neoliberal era: A study on the palliative care movement in Kerala. *Journal of Social and Economic Development*, *18*(1-2), 1-16.
57. Seamark, D., Ajithakumari, K., Burn, G., Devi, P. S., Koshy, R., & Seamark, C. (2000). Palliative care in India. *Journal of the Royal Society of Medicine*, *93*(6), 292-295.
58. Sengupta, J., & Chatterjee, S. C. (2013). Locating End-of-Life Care in Public Health Context: Is India Prepared for the Discourse? *Indian Anthropologist*, 43, 79-92.
59. Shabeer, C., & Kumar, S. (2005). Palliative care in the developing world: A social experiment in India. *European Journal of Palliative Care*, *13*, 76-79.
60. Shanmugasundaram, S., Chapman, Y., & O’Connor, M. (2006). Development of palliative care in India: An overview. *International Journal of Nursing Practice*, *12*(4), 241-246.
61. Singh, T., & Harding, R. (2015). Palliative care in South Asia: A systematic review of the evidence for care models, interventions, and outcomes. *BMC Research Notes*, *8*(1), 172 -187
62. Stjernsward, J. (2005). Community participation in palliative care. *Indian Journal of Palliative Care*, *11*(2), 111.
63. Stjernswärd, J. (2007). Palliative care: The public health strategy. *Journal of Public Health Policy*, *28*(1), 42-55.
64. Stjernsward, J., & Clark, D. (2003). Palliative medicine - A global perspective. In Doyle, D., Hanks, G.W., Cherny, N. and Calman, K.C. (Eds.) Oxford Textbook of Palliative Medicine, 3rd ed. Oxford University Press: Oxford, pp. 1199-1224. ISBN 9780198566984
65. Stjernswärd, J., Foley, K. M., & Ferris, F. D. (2007). The public health strategy for palliative care. *Journal of Pain and Symptom Management*, *33*(5), 486-493.
66. Suchitra, M. (2009). Kerala spearheads community-care health revolution. *Appropriate Technology*, *36*(2), 49.
67. Twycross, R. (2005). Death without Suffering? Plenary Lectures. *9^th^ Congress of the European Association of Palliative Care*, Aachen, Germany, 8-10 April 2005.
68. Vallath, N., Tandon, T., Pastrana, T., Lohman, D., Husain, S.A., Cleary, J., Ramanath, G., Rajagopal, M.R. (2016). [Civil-society driven drug policy reform for health and human welfare – India](https://www.ncbi.nlm.nih.gov/pubmed/28042065). *Journal of Pain Symptom Management*. 55, 518-532
69. Venkateswaran, C. (2003). *A Pilot Study of Factors Affecting Patient-Compliance to Curative Treatment of Cancer*. Unpublished Document.
70. Venkateswaran, C., & Kumar, T. M. (2006). Psycho-oncology in India: Emerging trends from Kerala. *Indian Journal of Palliative Care*, *12*(1), 34.
71. Venkateswaran, C., Jose, S., & Francis, A. P. (2014). Community mental health and NGO engagement: The Kerala experience. In Francis, A. (Ed.). *Social Work in Mental Health: Areas of Practice, Challenges, and Way Forward*, 276-300.
72. Vijay, D. (2012). *Collective Action Frame and Organizational Field Emergence in the Context of Palliative Care in Kerala, India*. Unpublished Dissertation. Indian Institute of Management Bangalore, India.
73. Vijay, D. (2018). Being Mortal: Beyond the Great Doctors, and What Matters in The End. <https://discoversociety.org/2018/02/06/being-mortal-beyond-the-great-doctors-and-what-matters-in-the-end/> Accessed on 1^st^ March 2018
74. Vijay, D., & Kulkarni, M. (2012). Frame changes in social movements: a case study. *Public Management Review*, *14*(6), 747-770.
75. WHO Cancer Pain Release. (1998). Calicut, India: A model for palliative care delivery**.** <http://www.whocancerpain.wisc.edu/?q=node/250> Accessed on 10^th^ March 2011.
76. WHO. (2007). *Cancer Control: Knowledge into Action*. Geneva, Switzerland: World Health Organization
77. Zaman, S., Inbadas, H., Whitelaw, A., & Clark, D. (2017). Common or multiple futures for end of life care around the world? Ideas from the ‘waiting room of history’. *Social Science & Medicine*, *172*, 72-79.
78. Zaman, S., Whitelaw, A., Richards, N., Inbadas, H., & Clark, D. (2018). A moment for compassion: Emerging rhetorics in end-of-life care. *Medical Humanities.* <http://dx.doi.org/10.1136/medhum-2017-011329>
