# Supplementary material for: Translation of a community palliative care intervention: Experience from West Bengal, India
Source: Wellcome Open Res. 2018 May 31;3:66. [Version 1] doi: 10.12688/wellcomeopenres.14599.1 (PMC6069742; doi:10.12688/wellcomeopenres.14599.1)
Supplement: Peer review history

# Peer review: Referee response for version 1

**DOI:** 10.21956/wellcomeopenres.15895.r33514

**Published:** 2018-07-30

**Authors:** Melanie Dembinsky

**Affiliations:** 1 Health Sciences & Sport, University of Stirling, Stirling, UK

The authors present a compelling institutional analysis of the translation of the Kerala community-based palliative care model to Nadia, West Bengal. Using comparative analysis, the contextual difference between the two locales are highlighted and how they impacted on the translation process. Drawing on current literature and their data collected over a period of 29 months study findings are presented in a clear and concise manner. This study is a valuable addition to the research literature on translation studies, and specifically in the field of healthcare interventions.

 Considering the importance given to patron-client relationships in Nadia, it is unclear where the researcher fit in within these relationships. How did the researchers navigate these relationships during data collection and how did their own community standing potentially impact on data collection?

*Overcoming low-legitimacy *is a key theme in the analysis, but it is unclear from the paper in which way the initial difficulties in legitimizing the project were overcome. The authors state that the symbolic power of the DM was crucial in overcoming initial reservations and hostilities about Sanjeevani, but it remains unclear why these were present in the first place if the entire conception and inauguration of the project was based on the initiative of the DM; wouldn’t this have been a key element highlighted to volunteers and patients alike?

 Evidence is given from the volunteers why they opted to engage with Sanjeevani in this form, but less has been said about the physicians and other medical staff who are crucial actors in the project’s foundation and implementation. What motivated them to become part of this community project and what are potential future obstacles in sustaining it when the support from physicians becomes less as Sanjeevani heavily relies on these sponsorships as described by the authors.

 The authors state that awareness and training workshops were held, but it remains unclear what form these took. Were the training workshops free to attend? How long did they last? How was a location for these workshops decided upon, and who could be a master trainer? Were training workshops’ organization and content taken from the Kerala model or specifically developed for the Nadia context?

 In summary, the article is a constructive addition to the research literature but could benefit from being more detailed in the presentation of the translation process. Acknowledging the importance of contextualization is a step in the right direction, and one healthcare interventions need to integrate in order to be potentially successful. However, to be able to successfully translate healthcare interventions to other geographical context it is necessary to be transparent about the processes involved in adjusting them to their local situations.

I have read this submission. I believe that I have an appropriate level of expertise to confirm that it is of an acceptable scientific standard.

# Peer review: Referee response for version 1

**DOI:** 10.21956/wellcomeopenres.15895.r33247

**Published:** 2018-07-30

**Authors:** Richard Harding

**Affiliations:** 1 Florence Nightingale Faculty of Nursing, Midwifery and Palliative Care, Cicely Saunders Institute, King’s College London, London, UK

This type of publication should be encouraged- novel models from LMIC and how they can be replicated are under-reported.

 ABSTRACT

- The 1 ^st^ sentence sounds as if Kerala created the first community palliative care model- I think the authors mean the model specific to Kerala?
- In results it would be helpful to be clear on how you mean by the “translator”.
- What is meant by solidarities here- a joining of different views of a set or common values coming together? What are “vertical solidarities”? These are really important messages for the community to use for replication so some of the language could be a little simpler? Given the importance, could you conclude with some recommendations?

 

 INTRODUCTION

- It would be really helpful to understand what has been lacking in prior palliative care “replication/transfer” studies- can you give some specific examples of what has worked what hasn’t and why? I’m not sure that I understand the sentence “the translator’s employment of symbolic power legitimates the nascent community form in this institutional context”.

Please describe your purposive sampling frame and also your sample characteristics. Please clarify which participant is giving data after each quote using an identifier.

 The results are very interesting and illuminating and clarify some of the statements in the abstract and introduction. It would be very helpful for readers if a set of recommendations could be provided?

I have read this submission. I believe that I have an appropriate level of expertise to confirm that it is of an acceptable scientific standard, however I have significant reservations, as outlined above.

# Peer review: Referee response for version 1

**DOI:** 10.21956/wellcomeopenres.15895.r33248

**Published:** 2018-06-14

**Authors:** Suhita Chopra Chaterjee, Tulika Bhattacharyya

**Affiliations:** 1 Department of Humanities & Social Sciences, Indian Institute of Technology Kharagpur, Kharagpur, India; 2 Indian Institute of Technology Kharagpur, Kharagpur, India

This is an interesting paper which examines the translation of Kerala’s community based palliative care form to Nadia, West Bengal. Emphasizing on the importance of ‘contexts’ in translational research, the authors provide an institutional analysis of Kerala and West Bengal which show marked differences between the two in terms of demographic indicators, legal infrastructure for palliative care and histories of civil-society organizing. A comparative analysis is used to identify aspects of the community form which have materialized in Nadia under major themes: resource mobilization, low levels of legitimacy, top down organizing, team and team leaders, occupational prestige and camaraderie. These themes were clustered according to interpretative categories. The findings emphasize a processual understanding of participation among frontline members, their meanings and interpretations. Three ‘key moments of translation’ are identified by the authors: the translator’s enactment of symbolic power (enacted by the District Magistrate), translating member relations by establishing a hybrid of vertical and horizontal solidarities which helped in resource mobilization and gaining organizational legitimacy, and translating social participation mainly through affective factors related to occupational prestige, status and camaraderie.                                             

 Despite the methodological rigour, the paper fails to provide a full spectrum of information needed to ensure replicability. It does not indicate steps taken to translate the Kerala model to suit local characteristics. For instance, what changes were made in mechanisms for developing knowledge, skills, tools and techniques, given the poor literacy of the population? How was good pharmaceutical management ensured in the absence of public health linkage? How were goals of palliative care achieved with limited access to morphine? And finally, how were sustainability issues addressed when funding structures were weak and when the power brokers, including majority of health professionals were ‘outsiders’? There is little information on nature of community participation in initial stages of programme planning (need assessment, monitoring, etc.) and information on their social practices and culture (see WHO, 2012).

 The importance of affective factors is noteworthy. However, this need not distract attention from rational-strategic planning necessary for judicious choice of a locale for translation. One wonders why in the absence of situational factors considered crucial to the case success of Kerala, Nadia was chosen, at all. Again, there is very little to help readers to gauge the success of the translation process being described. Mere information on increasing coverage, mobilization and volunteer strength does not adequately reflect on nature of palliative care delivery outcomes. In fact, the widely acclaimed success of the Kerala model itself is unsubstantiated by large scale impact assessments. Although one does not wish to  question its success or its  huge contribution  to community initiatives in palliative care, two micro investigations bring out glaring gaps in home based palliative care services (Jayalakshmi *et al*., 2016 ^1^) and in policy implementation as well (Jayalakshmi & Chatterjee, 2017 ^2^). It is the moral responsibility of the translators to recognize the gaps before the zeal to translate takes over.

 The paper ends up by reiterating the very obvious (at least, in mainstream sociological literature) namely, the contested nature of communities, problems in decontextualised transfer models, and importance of prospectively analyzing institutional contexts.

We have read this submission. We believe that we have an appropriate level of expertise to confirm that it is of an acceptable scientific standard.

## References

[1] JayalakshmiRChatterjeeSCChatterjeeD: End-of-life Characteristics of the Elderly: An Assessment of Home-based Palliative Services in Two Panchayats of Kerala. *Indian J Palliat Care*.22(4) : 10.4103/0973-1075.191857 491-498 10.4103/0973-1075.191857 27803573PMC5072243

[2] JayalakshmiRSuhitaCC: Home-based Palliative Services under Two Local Self-government Institutions of Kerala, India: An Assessment of Compliance with Policy and Guidelines to Local Self-government Institutions. *Indian J Palliat Care*.23(1) : 10.4103/0973-1075.197947 65-70 10.4103/0973-1075.197947 28216866PMC5294441
